# Validating explicit rating tasks for measuring pronunciation biases: A case study of ING variation

**DOI:** 10.3758/s13428-026-02952-y

**Published:** 2026-02-17

**Authors:** Aini Li, Meredith Tamminga

**Affiliations:** 1 Department of Linguistics and Translation, City University of Hong Kong, 83 Tat Chee Avenue, Hong Kong SAR, HK China; 2 Department of Linguistics, University of Pennsylvania, 3401-C Walnut Street, Philadelphia, PA USA

**Keywords:** Word bias, Phonological variation, User estimates, Methodological validation, Sociolinguistic knowledge

## Abstract

Spoken language is highly variable, as words can have different pronunciation variants. A growing body of psycholinguistic research has employed experimental methods such as explicit rating tasks to obtain user biases toward different pronunciation variants. However, no prior work has empirically validated whether experimentally elicited user estimates accurately reflect real-world usage patterns. By correlating user estimates and conversational speech data for English variable ING pronunciations under different experimental prompts, we found that while rating tasks can provide word biases that do correlate significantly with corpus word biases, the correlations are only modest and there are asymmetries in the relationship between elicited word biases and corpus word biases. These findings call for future research to incorporate word biases into the study of sociolinguistic variation and language processing.

## Introduction

Pronunciation variability is a fundamental feature of spoken language, with words often exhibiting multiple pronunciation variants. For instance, in American English, the word WORKING can be pronounced variably as *working* or *workin’*. Among many factors that influence such variable pronunciation, language users may have word-specific tendencies for the rates at which they choose one pronunciation variant over another. For example, speakers may be more likely to say *studying* than *studyin’*, but more likely to say *kickin’* than *kicking*. For brevity, we will refer to this type of word-specific variant bias as “word bias”.

This concept of word bias is not merely a feature of language use; it is a critical component in understanding how listeners perceive, process, and produce speech. For example, research in psycholinguistics and sociolinguistics suggests that these biases are relevant to the mental representation of words and the cognitive processes underlying sociolinguistic perception (Bürki et al., 2010; Connine, 2004; Li, 2024). Consequently, to investigate the role of word bias in these domains, researchers will need reliable methods of estimating these word-specific tendencies. Traditionally, researchers have derived word bias estimates from large speech corpora, as these data reflect real-world usage patterns (e.g., Connine, 2004). However, this approach is often highly resource-intensive, requiring significant time and effort for data collection, transcription, and annotation. Furthermore, corpus-based methods are frequently hampered by data sparsity, where low-frequency words or linguistic features yield too few examples for robust statistical analysis. An alternative, less laborious approach involves using experimental tasks, such as rating tasks, to elicit judgments about pronunciation variants directly from language users (e.g., Bürki et al., 2010). This method enables researchers to target the variable in question explicitly and efficiently gather data on a large number of words.

However, for these elicited estimates to be a viable tool, a crucial assumption must hold: they should accurately reflect the production patterns observed in natural speech. The validity of using subjective ratings as a proxy for objective usage patterns cannot be taken for granted. To the best of our knowledge, the fundamental link between word bias estimates elicited from experiments and word bias estimates extracted from corpora has not yet been systematically established. This methodological gap limits the interpretability and utility of using experimental measures in both psycholinguistic and sociolinguistic work. As a result, effects attributed to word bias from elicited measures may simply reflect participants’ metalinguistic judgments rather than actual production patterns.

Given this empirical research gap, we present a methodological validation study that evaluates the efficacy of rating tasks for approximating real-world biases toward pronunciation variants for specific words, using the sociolinguistic alternation between*-ing* and*-in’* pronunciations as a test case (we will use all-caps ING to refer to the alternation). Specifically, we ask three research questions:

- RQ1: Does an explicit word-bias rating task produce word-bias estimates for ING that correlate with word-bias estimates calculated from corpus data?
- RQ2: If so, do we observe stronger correlations if we prompt participants to reflect on their expectations about community-level patterns, or on their own production patterns?
- RQ3: Does providing minimal syntactic context to resolve part-of-speech ambiguity improve the correlation between elicited and corpus estimates?

Our results demonstrate that while rating tasks can provide word biases that do correlate significantly with corpus word biases, the correlations are only modest and there are asymmetries in the relationship between elicited word biases and corpus word biases. This suggests that such rating tasks should be treated with caution, raising important questions about the cognitive processes involved when people rate single-word prompts in rating tasks of a similar nature. Additionally, our two prompts targeting individual versus community-level expectations/judgments yielded similar results in these explicit rating tasks.

This validation has both methodological and theoretical value. First, our findings suggest that rating tasks can offer a practical proxy for usage data due to their ease of implementation, particularly when compiling large corpora is impractical or yields sparse token counts for low-frequency linguistic features. However, these tasks should be viewed as a useful supplement to, rather than a complete replacement for, corpus data; their imperfect correlation with real-world usage should be kept in mind, especially if word-specificity is a key question under investigation rather than simply a control predictor.

Second, our results indicate that individuals do have at least some degree of implicit knowledge of word bias for probabilistic pronunciation alternations, and can report these biases in experimental settings. This aligns with work suggesting that the mental representation of word bias is a productive target of inquiry in its own right to better understand the perception and production of sociolinguistic variation (Bürki et al., 2010; Li, 2024). To facilitate future research, we also provide elicited word biases for a total of 318 ING-containing words in English that have been tested in our study.

As an overview, this paper is structured as follows. Section “Background” reviews prior work on the role of word-level factors in language processing and sociolinguistic variation in general. Methods adopted in this study are detailed in Section “General methodology”. Section “Analysis and results” reports our main analyses and results. Section “Experiment 2: Community-level expectations vs. Individual language use” and Section “Experiment 3: Context matters for elicited word biases” compare the results from different prompts we tested, highlighting the role of syntactic context in eliciting word biases. Section “General discussion” discusses the implications of our findings and concludes the paper.

## Background

### Word-specific variability in language processing

We define word bias as a systematic preference for particular pronunciation variants to occur more frequently with specific words. These preferences may stem from a mix of linguistic or social factors, such as frequency, morphological structure, collocational patterns, or symbolic/ideological associations with particular speaker groups (Fischer, 1958; Forrest, 2015; Tagliamonte, 2004). Its multidimensional nature makes empirical measures of word bias a valuable venue for research across different theoretical approaches, even when their underlying mechanisms are not fully understood.

One well-studied aspect of word bias is frequency effects, where high-frequency words often lead to sound changes like reduction or lenition (e.g., coronal stop deletion in English) (Bybee, 2002; Clark & Trousdale, 2009; Dinkin, 2008; Phillips, 1984; Pierrehumbert, 2006). Beyond frequency, other factors such as stylistic register, phonetic environment, morphological structure, word length, and stress may also shape variant preferences (e.g., Cofer, 1972; Forrest, 2015; Kendall, 2010; Labov, 2006 [1966]; Trudgill, 1974). For example, it has been suggested that formal verbs—ones that are used in formal situations or have more formal connotations (e.g., *studying*)—may show stronger preferences for more formal variants, while informal verbs favor less formal variants (Fischer, 1958). Crucially, Vaughn and Kendall (2018) found that individuals were able to self-report how surprising they would find it to hear different sentences with different pronunciation variants (e.g., *working* vs. *workin’*) and that their ratings aligned with broad grammatical patterns of variable ING production (e.g., three-syllable pronouns and adjectives showed significant rating differences). However, some finer grammatical distinctions that have been found in corpus data (e.g., gerunds vs. progressives or two-syllable pronouns) were not reflected in participants’ ratings. This raises the question of whether fine-grained word-specific biases can be picked up with a rating task.

Word-level variability has attracted growing interest in psycholinguistic research, particularly in relation to word perception and production (Cutler, 2012). For example, Connine (2004) investigated how English listeners recognize phonological variants involving schwa deletion (i.e., *corporate* $\rightarrow$ *corp’rate*). They examined whether the surface frequency of each variant (schwa deleted or retained) influenced syllable-counting and lexical decision speed-up. Using corpus data, they compared words with high vs. low schwa-deletion rates and found that words with high deletion rates elicited faster responses. This suggests that the frequency with which a given variant was experienced in a particular lexical context significantly influences how phonological variants are processed. Similarly, Ranbom and Connine (2007) demonstrated that the production frequency of nasal flap variants in American English (based on corpus statistics) affected lexical decision accuracy and speed, with words that favor nasal flap being recognized faster and more accurately than those with a lower nasal flap frequency. Pitt et al. (2011) extended these findings by showing that variant recognition patterns could be predicted by word biases. Other than the effects of word-specific variability in speech perception, such effects have also been documented in speech production. For instance, Bürki et al. (2010) looked at the role of the relative frequency of the two pronunciation variants (the schwa variant vs. the reduced variant) in French schwa words in picture naming, and found that variants with higher relative frequencies were produced faster compared to variants with lower variant relative frequencies, suggesting that lexical activation in word production is sensitive to word-specific contexts.

While word bias has come into greater focus as a predictor of word processing, studies have differed in the methods used to operationalize word biases. Some studies use corpus data as the source of word biases (Connine, 2004; Ranbom & Connine, 2007). Others have developed laboratory measures to elicit word biases. For example, Bürki et al. (2010) used an experimental rating task to estimate how likely each variant is to occur in a given variable-schwa word, with the ratings collected being taken as proxies for word biases. Similarly, Pitt et al. (2011) used a memory-demanding production experiment where participants memorized a sentence, integrated a given word into it, and produced a new sentence. The goal of this method was to elicit casual speech so as to estimate the frequency of different variants in different word contexts. Given these different methods, we ask whether elicited word biases are capturing the same phenomenon as the naturalistic corpus word biases.

With this background in mind, the present study aims to offer a methodological validation for these methods. Specifically, we elicit word biases through a rating task, loosely modeled on that used in Bürki et al. (2010), to collect individuals’ estimates of word biases. We then correlate these elicited word biases with estimates from corpus data. The particular type of word we use as a testbed is the English variable ING, which will be introduced in more detail in the follow-up section.

### English ING variation

The English variable ING is a recognizable and well-studied sociolinguistic feature. We use all-caps "ING" to refer to the probabilistic alternation between word-final /ŋ/ and /n/ after unstressed /ɪ/, as in *thinking* $\sim$ *thinkin’*. Monosyllabic words that do not have an unstressed /ɪ/ (e.g., “sing”, “wing”) do not participate in the alternation. For the last few decades, extensive work has been done in quantitative sociolinguistics to document how ING in conversational speech is conditioned by various *social* (e.g., speakers’ socio-economic class, gender, race, and regional dialects), *linguistic* (e.g., phonological and morphological environments) and *cognitive* factors (Abramowicz, 2007; Campbell-Kibler, 2007, 2011; Cofer, 1972; Fischer, 1958; Forrest, 2015; Hazen, 2008; Houston, 1985; Kiesling, 1998; Labov, 2006 [1966], 2001; Tagliamonte, 2004; Tamminga, 2016; Trudgill, 1974; Wagner, 2013; Wagner & Sankoff, 2011; Wald & Shopen, 1985). Among the two variants*-in’* and*-ing*, the former is considered non-canonical or informal whereas the latter is considered canonical or formal (Labov, 2001).

The choice between*-ing* and*-in’* in conversational speech may also exhibit word-specific preferences that are not clearly reducible to the kinds of factors noted above. In an influential early study, Fischer (1958) proposed that verbs that are markedly ‘formal’ in meaning or register associations tend to be suffixed with the formal*-ing* variant (e.g., words like *criticizing*, *reading*, and *visiting*), whereas verbs that are considered ‘informal’ are more likely to be followed by the informal*-in’* variant (e.g., words like *flubbin’* and *hittin’*). These word-specific differences in the use of ING may be partially driven by different word frequencies, but at the same time seem to go beyond frequency. However, no systematic investigations have been carried out of word-specific stylistic tendencies towards*-ing* or*-in’*.

ING is chosen as the test case for this study not only because it is one of the most well-studied sociolinguistic features in the quantitative sociolinguistics literature, as demonstrated above, but also due to these word-specific tendencies that have been observed for this feature in corpus data, suggesting that people may have intuitions around these word biases for ING. The availability of existing corpus data, coupled with the prior empirical study (i.e., Vaughn and Kendall (2018)) showing that we have evidence for self-reported judgments that capture one aspect of how ING is used in production, makes our methodological validation feasible.

## General methodology

### Goals

The present study examines whether elicited word biases correlate with corpus word biases. Inspired by the task adopted in Bürki et al. (2010), our study uses a rating task to elicit individuals’ ratings of ING variants associated with specific words. We refer to this metric as word bias, i.e., the probability of*-ing* (as opposed to*-in’*) given a specific word. The goals of this present study are threefold: First, we aim to elicit word biases for variable ING through a rating task to collect users’ estimates. Using these users’ estimates, we then examine whether these elicited word biases correlate with word-specific*-ing* probabilities extracted from conversational corpus data (Experiment 1). Second, to get at the type of elicited word biases that seem most reliable, we also compare the strengths of the correlations using word biases elicited with two different prompts: one targeting users’ expectations about community-level patterns and another targeting users’ own production. This comparison is motivated by the possibility that participants may have different intuitions about what they hear around them versus their own language use, which the task instructions might pick up on. To be specific, our first prompt “Which are you more likely to **hear**" directs people to reflect on their expectations about the community-level patterns, and our second prompt “which are you more likely to **use**" directs people to reflect on their own production. By comparing the correlations using word biases elicited from different questions, we can move toward best practices for future research using similar types of tasks (Experiment 2). Third, we address potential ambiguities in the stimuli by introducing syntactic context, and ask whether this improves correlations between elicited and corpus word biases (Experiment 3).

### Materials

Corpus word biases of ING-containing words were taken from Tamminga (2014), which were originally generated using the Philadelphia Neighborhood Corpus (PNC), a collection of transcribed and aligned sociolinguistic interviews that were originally conducted in Philadelphia, USA between 1973 and 2010 (Labov & Rosenfelder, 2011). In Tamminga (2014), 108 interviews with white Philadelphians were selected from the PNC, and all words containing variable ING were manually coded for which variant the speaker used in that instance, as well as the word’s grammatical category. From these words, we selected those with at least 20 occurrences, assuming that speakers have more reliable estimates for higher-frequency items (*N* = 68). These 68 words served as the real-world corpus word biases for testing the correlation between corpus and elicited word biases. It is important to note that the corpus data represents the speech of a particular time and place in American society; while factors like dialect and social class are certainly expected to influence ING rates overall, we do not know of any reasons that social factors would systematically influence relative word biases. That is, for the most part, we think that an*-ing*-variant-favoring verb in Philadelphia would also be an*-ing*-variant-favoring verb in New York, or Atlanta, or Detroit. This would also necessarily be an issue with any corpus data we chose for comparison.

For the rating task, ING-containing words drawn from two different sources were included as critical stimuli. The first set comprised the 68 words selected directly from PNC. In addition, to ensure our stimuli covered a broad range of lexical properties beyond those found in conversational speech, we supplemented this set with 250 ING-containing words from the large-scale stimulus list compiled by White (2021). The inclusion of those 250 words from White (2021) was also intended to create a dataset of elicited word biases for variable ING for future researchers to use. The stimulus list in White (2021), to the best of our knowledge, offered one of the most comprehensive and recent compilations of English ING words that have actively been used in psycholinguistic and sociolinguistic research (e.g., White et al., 2024). In the end, 318 words were selected and served as words to be rated in Experiment 1 and Experiment 2. Only words with corpus word biases (*N*=68) were used in Experiment 3 for reasons that we detail below.

In Experiment 1 and 2, all the 318 words were divided into five lists, each containing around 63 ING words. Within each list, three catch trials were included to ensure that participants were paying attention and doing the task as expected. These catch trials consisted of ratings for monomorphemic words with non-variable ING: *bring*, *string* and *ring*. If participants did not strongly prefer *bring* over *brin’*, it could indicate inattention, as *brin’* is ungrammatical and should not be rated as a well-formed option. In Experiment 3, participants rated all 68 words with catch trials in one list.

**Fig. 1:**
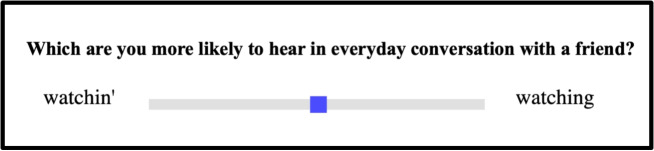
Experiment 1: Experimental interface for the word-specific variant bias rating task

### Procedure

A word bias rating task was designed and administered online through PCIbex, a platform that uses the PennController experiment toolkit for running experiments online (Zehr & Schwarz, 2018). The rating task was adopted across the three experiments. After giving consent, participants were instructed to use the slider on a slider bar to indicate which pronunciation of a given word they were more likely to hear in everyday conversational speech with their friends, as illustrated in Fig. 1. Participants needed to drag the slider left or right to indicate their pronunciation preferences for each word that was presented. In the instructions, participants were explicitly told that the more likely they were to choose one pronunciation over another, the further they should drag the slider. An example was provided to participants for illustrative purposes before they proceeded to complete the task. Because ING variation is highly dependent on speech style, participants were instructed to indicate which pronunciation they were more likely to hear in daily conversations with friends. This ensured that ratings reflected comparable social contexts across words (Campbell-Kibler, 2011). To direct participants to reflect on community-level patterns, we used the prompt “Which are you more likely to **hear** in everyday conversation with a friend" (Experiment 1). To direct participants to reflect on individual-use patterns, we used the prompt “Which are you more likely to **use** in everyday conversation with a friend" (Experiment 2). To test the role of syntactic context, we used the same prompt as Experiment 2 in Experiment 3.

After the rating task, participants were asked to fill out a questionnaire through which they shared their demographic information, including their age, gender, ethnicity, the country they spent most of their early childhood in, as well as any languages other than English that they speak. The whole rating task took around 10 min to complete.

## Analysis and results

### Experiment 1: Correlations based on community-level expectations

To address the first research question, i.e., whether elicited word biases of*-ing* (vs.*-in*) are correlated with how the two variants are used in conversational speech, we conducted a correlation analysis using the results from the rating task, with a focus on the 68 words about which we have both elicited and corpus word biases.

**Fig. 2:**
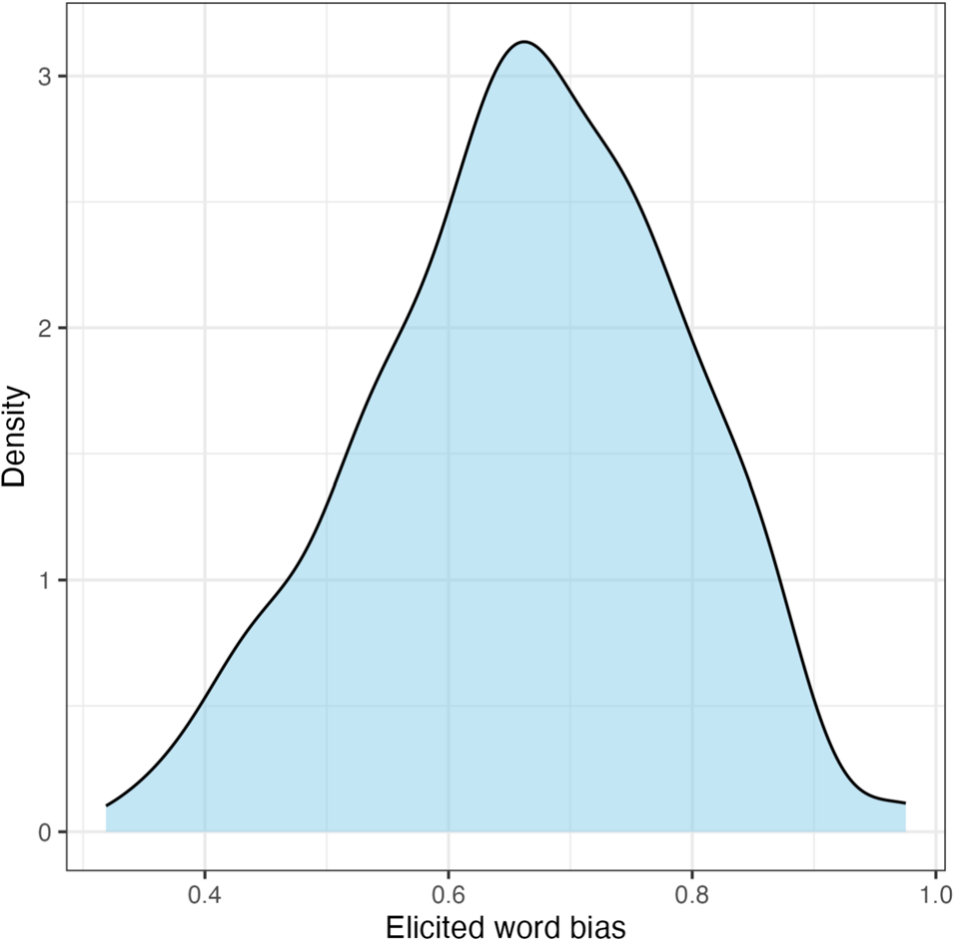
Experiment 1: Distribution of elicited word biases

**Fig. 3:**
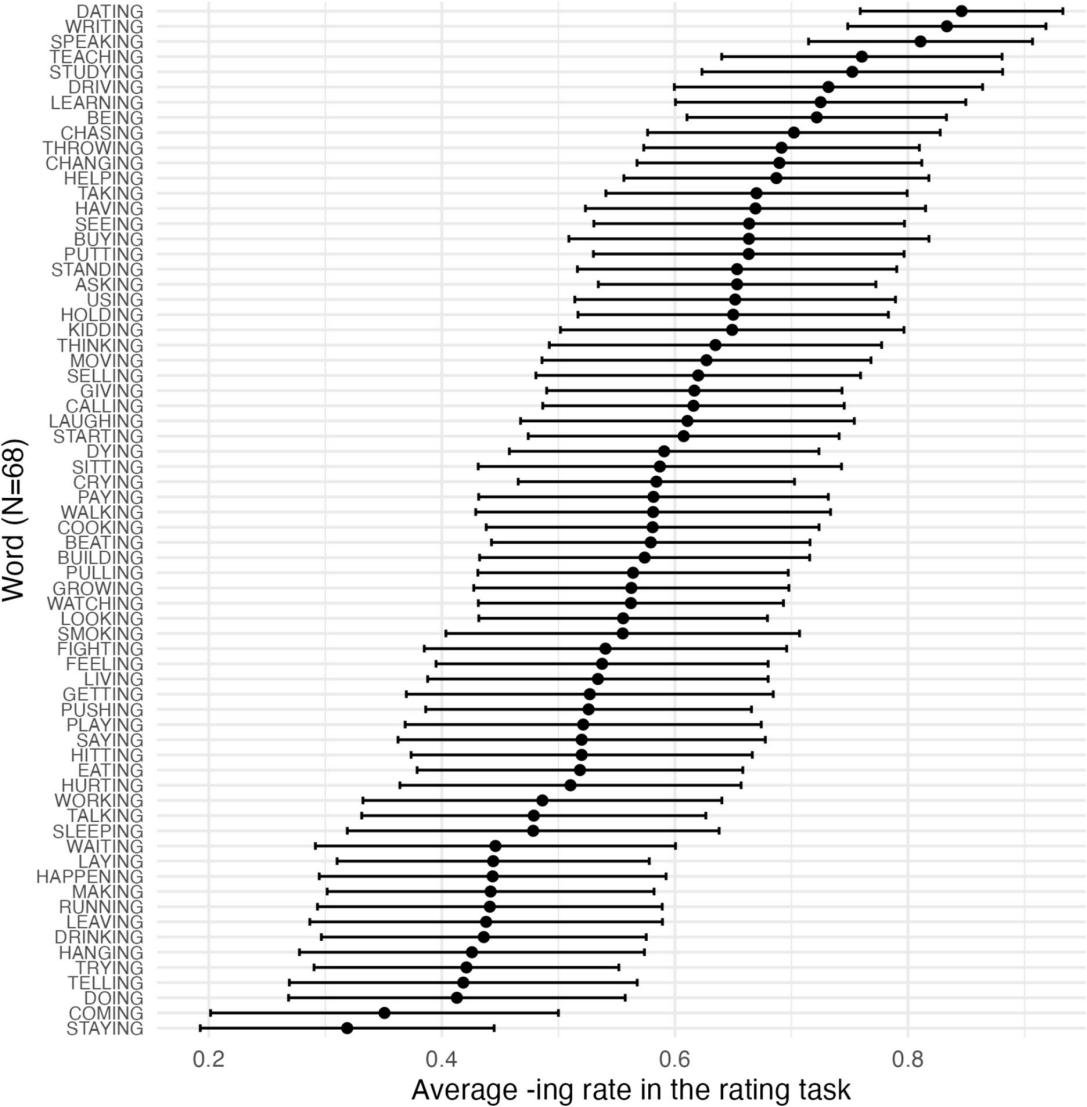
Experiment 1: Word-specific confidence intervals for the 68 ING words taken from PNC

#### Participants

A total of 150 self-reported monolingual American English speakers (73 women, 74 men, and three other) were originally recruited from Prolific in return for US$2, with approximately 30 participants being included in each of the five lists. Therefore, each word was rated by around 30 participants. This sample size of 30 ratings per word was selected to be comparable with sample sizes used in similar norming studies (Warriner et al., 2013). Among these participants, six were aged between 17 and 20 years old, 46 were aged between 21 and 25 years old, and 99 were over 31 years old. In terms of race and ethnicity, there were two Asians, 23 African Americans, 116 Caucasians, two Hispanics, five people of mixed race, and one Native American.

#### Analysis and results

Slider-bar positions were converted to real numbers ranging between 0 and 1: the larger the number, the stronger the tendency to choose the variant*-ing* for a given word. Before any analysis was conducted, participants who did not pass catch trials were excluded (*N* = 25). Figure 2 shows the raw distribution of elicited word biases for 318 variable ING words.

Elicited word biases were then extracted from the rating task for the 68 ING-containing words that were originally taken from PNC. Figure 3 plots the mean rating and 95% confidence interval for each of the 68 words. We can see from the graph that there are word biases for ING: some words are more likely to be rated as favoring*-ing* and some words are more likely to be rated as favoring*-in’*. However, the width of the confidence intervals indicates that there is still substantial variability or uncertainty in the ratings for any given word.

**Fig. 4:**
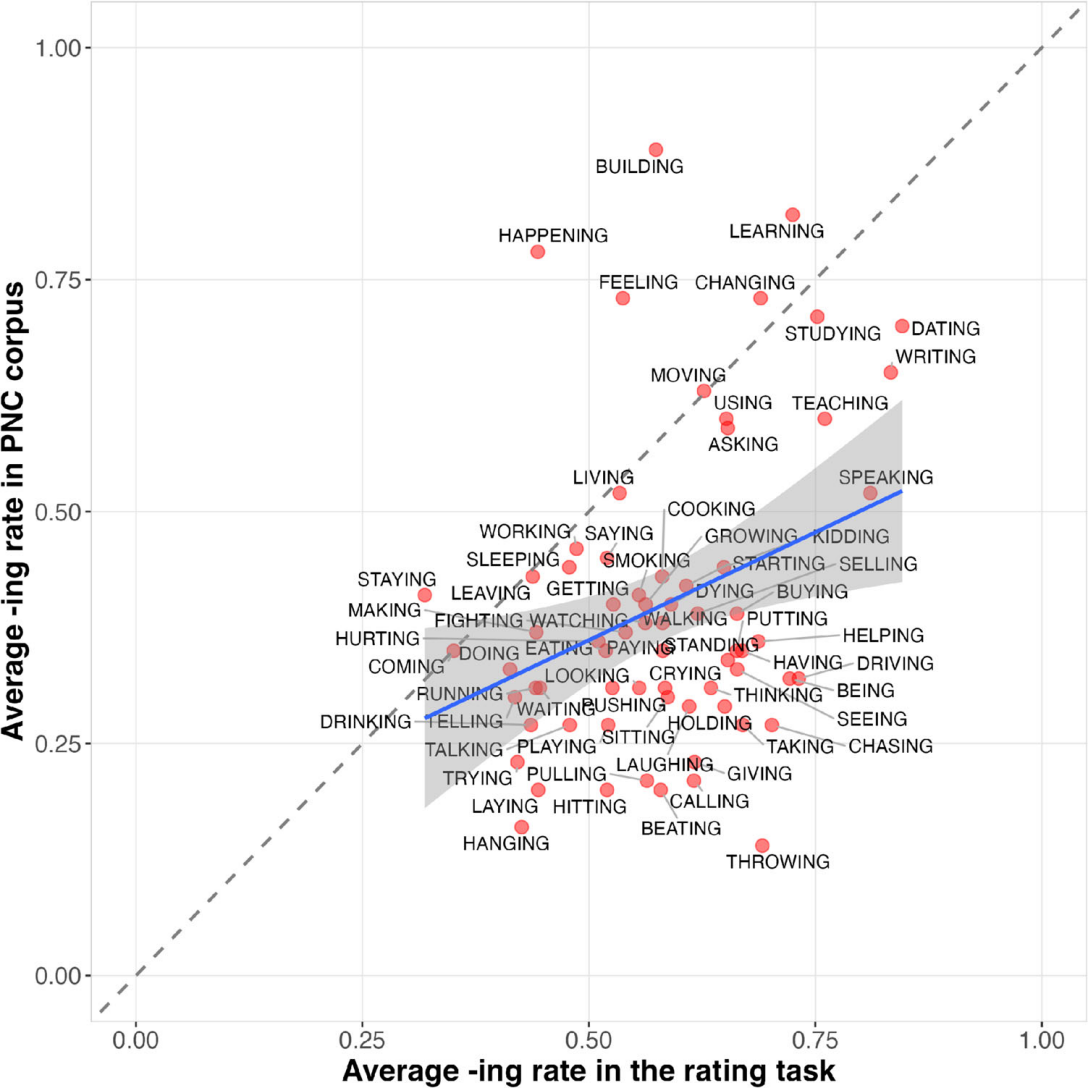
Experiment 1: The correlation between corpus word bias of ING and elicited word bias of ING across different word types

A Pearson correlation test was used as a measurement to test the correlation between participants’ preference for*-ing*-containing pronunciations, as opposed to their*-in’*-containing counterparts, with the actual use of the*-ing* variant (henceforth corpus word bias) for a given word in conversational speech. Using ratings from 125 participants, we found a significant, though modest, positive linear correlation between the corpus word biases and their elicited word biases (Pearson’s R = 0.315, *p* < 0.001). This correlation is further illustrated in Fig. 4. Figure 4 plots the correlation between elicited word bias of*-ing* in the explicit rating task and the corresponding corpus word bias of*-ing* for the same words. Take the word TEACHING as an example. The rate at which the word TEACHING is pronounced as *teaching* instead of *teachin’* is around 60% in our conversational speech data. In the rating task, the elicited preference for *teaching*, as opposed to *teachin’*, was 75%, which is comparable with the corpus word bias.

However, a closer inspection of the data distribution as shown in Fig. 4 reveals some asymmetries that bear further consideration. For words that favor*-in’* in the corpus, there is a wide range of ratings, weakening the correlation; whereas for words that favor*-ing*, ratings appear more consistent. Additionally, the data points fall disproportionately to the right of the x=y line, indicating that elicited word biases are higher than corpus word biases overall. This may reflect an unsurprising global bias toward the normative standard/canonical variant, either in general or in an experimental setting.

**Fig. 5:**
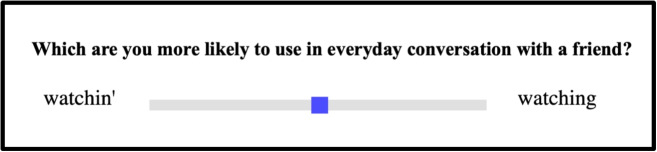
Experiment 2: Experimental interface for the word-specific variant bias rating task

#### Interim discussion

The results of Experiment 1 demonstrate that when being asked to indicate the relative frequency of*-ing* (vs.*-in’*) for a given word, individuals can broadly distinguish which words are more likely to favor the*-ing* variant compared to others, but elicited word biases are noisy and imperfect. However, a question remains regarding the nature of the prompt. Experiment 1 asked participants what they were likely to *hear*, targeting community expectations. It is possible that prompting participants to reflect on their own language use might yield different, or perhaps more accurate, elicited biases. To test this, we conducted Experiment 2.

### Experiment 2: Community-level expectations vs. Individual language use

Although word biases elicited through the rating task can reflect patterns in conversational speech, does prompting participants to reflect on their own language uses—rather than community-level patterns—make a difference? To test this, we repeated the rating task with the same materials (i.e., 318 ING-suffixed words in five lists) and the same setup as in Experiment 2.

#### Participants

To keep the same standard for our sample size, a total of 176 participants (88 women, 83 men and five other) were originally recruited from Prolific to participate in the second version of this rating task (35 participants in each of the five lists). These participants did not participate in Experiment 1 and were compensated for their participation with US $2. Among them, seven were aged between 17 and 20 years old, 52 were between 21 and 25 years old, and 117 were over 31 years old. There were no Asians, ten African Americans, 147 Caucasians, five Hispanics, 12 people of mixed race, one Native American, and one unspecified.

All the participants followed the exact same procedure as described in Section “General methodology” except that participants in Experiment 2 were given a slightly different prompt, as illustrated below in Fig. 5. The word **hear** in the previous version was changed to the word **use**, aiming to elicit ratings that target individual use of word-specific*-ing* biases.

#### Analysis and results

**Fig. 6:**
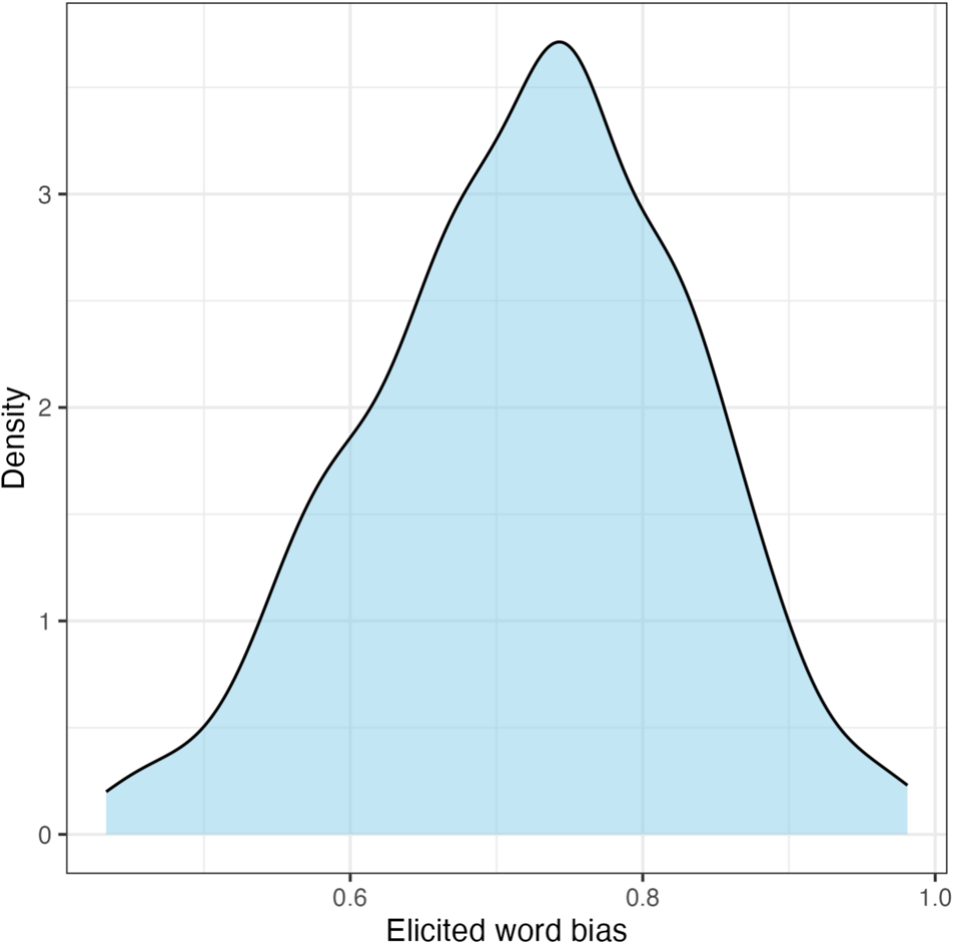
Experiment 2: Distribution of elicited word biases

**Fig. 7:**
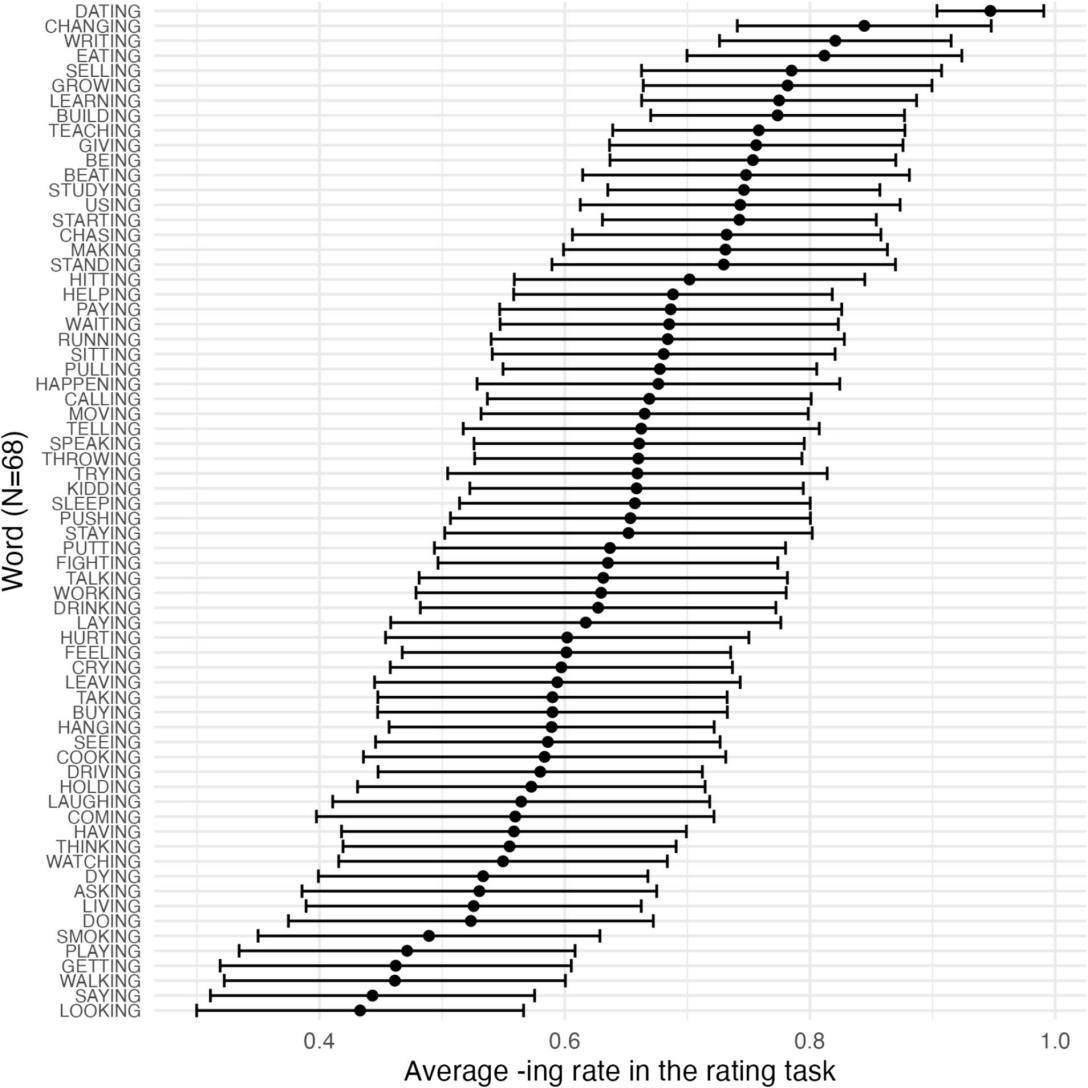
Experiment 2: Word-specific confidence intervals for the 68 ING words taken from PNC

Following our previous practice, we first excluded participants who did not pass the three catch trials (*N* = 21). This left data from the remaining 155 participants to be included in the correlation analysis. Figure 6 shows the raw distribution of elicited word biases for 318 ING words under the new prompt. Figure 7 again plots the mean rating and 95% confidence interval for each of the 68 words used in Study 2.

**Fig. 8:**
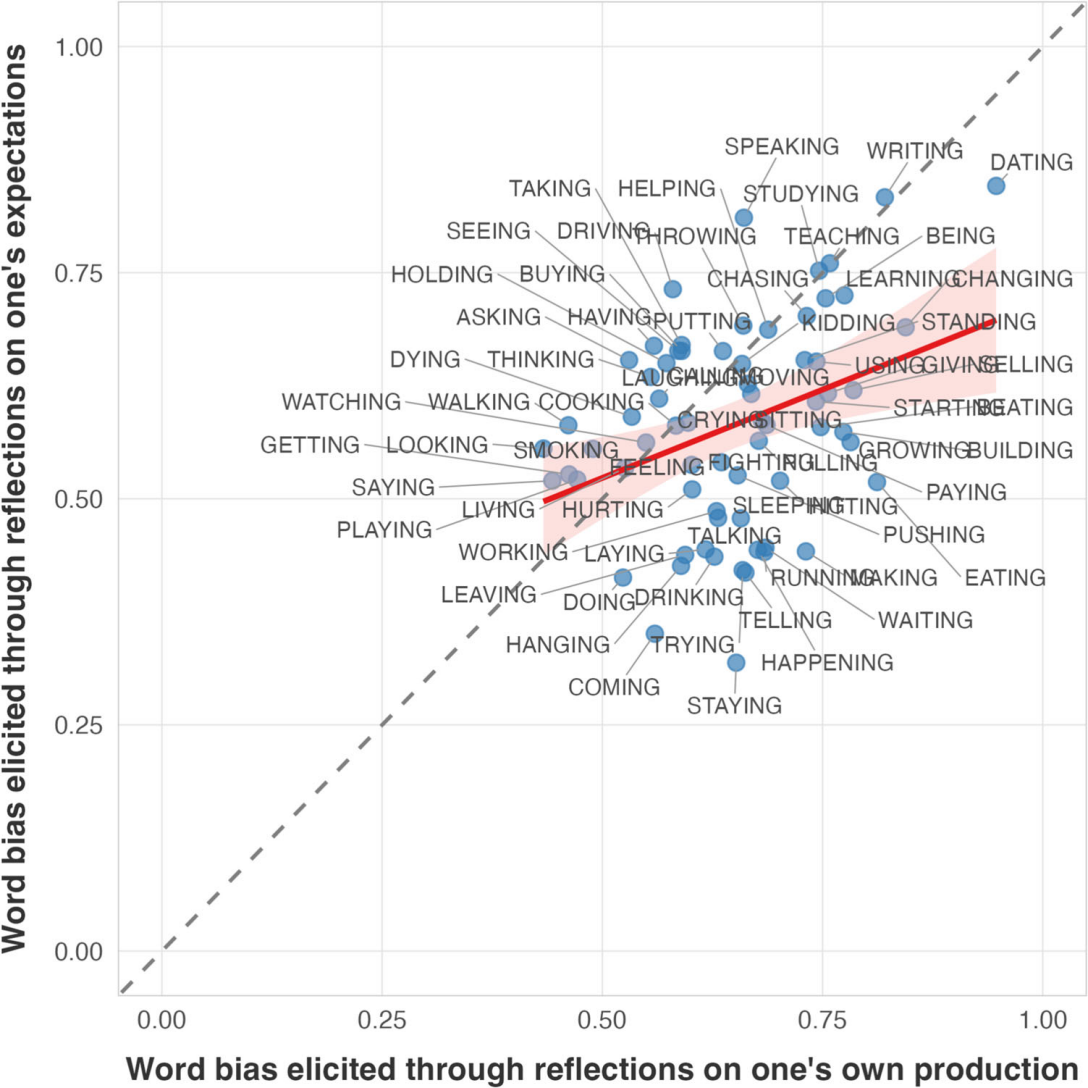
Correlations between word biases elicited through the two different prompts

A Pearson correlation test was conducted based on the 68 ING-suffixed words that were extracted from PNC. The results again found a significant positive linear correlation with modest strength between self-reported word-specific*-ing* biases and the actual word-specific*-ing* biases in the corpus (Pearson’s R = 0.318, *p* < 0.001). Crucially, the two biases elicited under different prompts—one targeting one’s own production patterns and another targeting one’s expectations—are also highly correlated (Pearson’s R = 0.36, *p* < 0.01), as can be seen from Fig. 8.

We further compared the two dependent Pearson correlations (i.e., correlation between elicited word biases under the prompt emphasizing community-level expectations and corpus word biases versus elicited word biases under the prompt emphasizing individual-level use and corpus word biases) using the cocor package in R (Diedenhofen & Musch, 2015), which tests for significant differences between overlapping correlations from the same sample. We found no significant difference in their correlation strengths (p>0.05). This suggests that the values elicited are very similar in both versions of the rating task, suggesting that the different wordings we tested do not influence the ratings.

#### Interim discussion

By correlating word biases obtained through the rating task and ING use in the corpus data, what has been established so far is that individuals are able to introspectively reflect on their expectations or own production and report the word biases of ING that are roughly consistent with the actual use of this variable in conversational speech. It is worth noting, though, that some of the highest*-ing* rates exhibited through both the rating task and the conversational speech are nouns and adjectives (see Fig. 4). These grammatical categories are well known to favor*-ing* (Houston, 1985). This suggests that the significant correlation may be driven by the overall grammatical (i.e., morphological) conditioning of ING, rather than purely word-specific biases. Thus, we next ask whether the correlation persists when the grammatical category is controlled for. For these follow-up analyses, we used the prompt asking participants to reflect on their own production patterns, since it had the slightly higher correlation with the corpus data in Section “Experiment 2: Community-level expectations vs. Individual language use”.

**Fig. 9:**
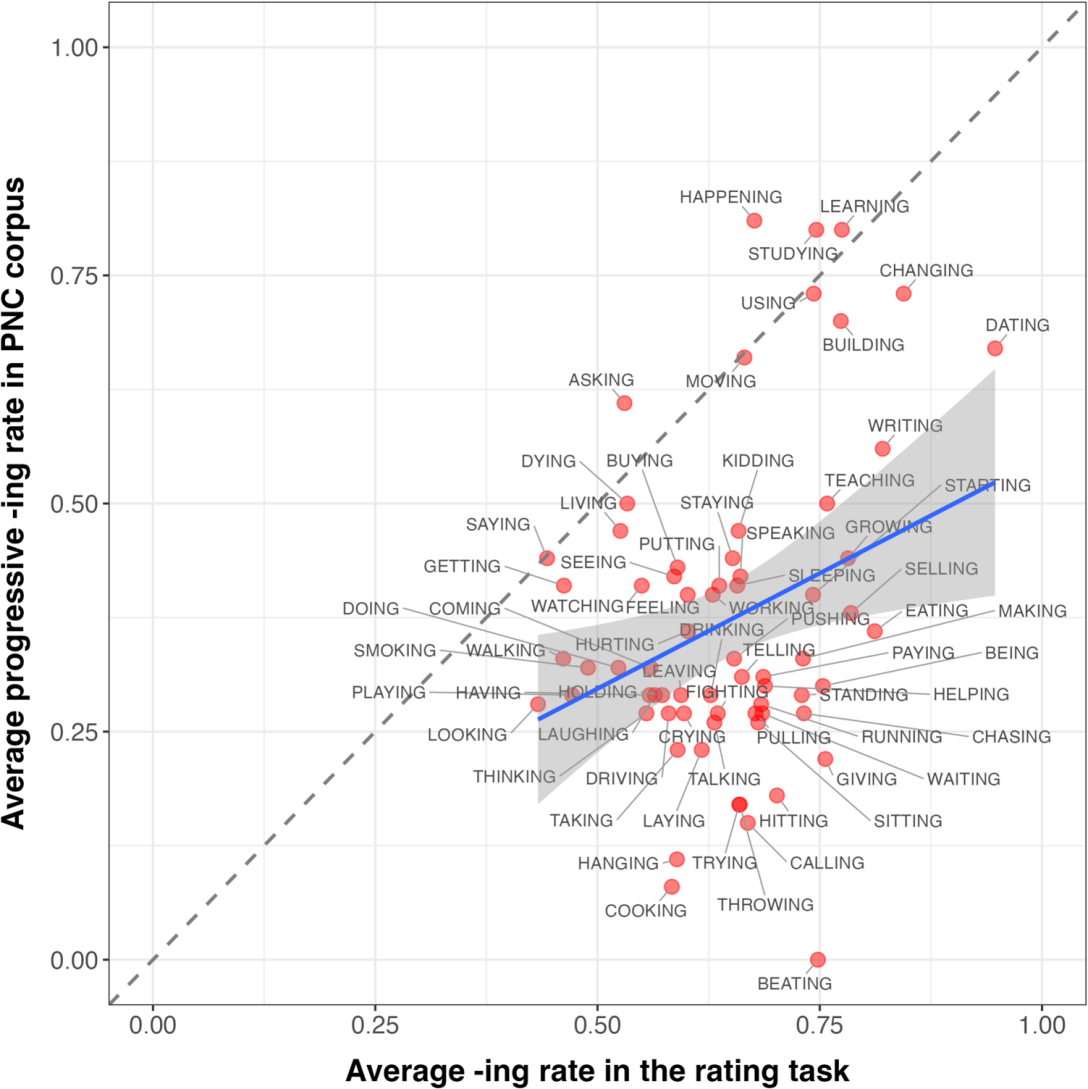
Correlations between corpus word bias of ING and elicited word bias of ING for progressive words only

To isolate elicited word biases independent of different parts of speech, we recalculated the correlation by including only corpus observations where an*-ing* word functioned syntactically as a progressive verb (the category that makes up 65% of the word tokens in the data). This means we did not exclude word types entirely; for example, a word like *building* was included in this analysis when it occurred as a verb but excluded when it occurred as a noun. The correlation between the average*-ing* rate in the rating task and the average*-ing* rate in PNC when ING words function as progressives is further plotted in Fig. 9. Pearson correlation analysis again revealed a positive correlation for progressive verbs (cf. Pearson’s R = 0.32, $p<$ 0.01 for overall *ing* rate correlation and Pearson’s R = 0.30, $p=$ 0.01 for progressive*-ing* rate correlation). This confirms that the initial positive correlation between elicited and corpus word biases is not solely driven by word categories with strong*-ing* bias (e.g., monomorphemes).

However, the grammatical function of a word is identifiable from corpus data but is ambiguous in the perception data from our rating task^1^. For instance, participants could interpret words in isolation, like BUILDING or BEATING as either a noun or a verb, which could affect their rating. Therefore, to address this source of ambiguity, we performed a follow-up rating task (Experiment 3).

### Experiment 3: Context matters for elicited word biases

To address the ambiguity of grammatical category present in Experiments 1 and 2, Experiment 3 introduced a minimal syntactic context that included an auxiliary verb “was” (e.g., rating “was *building*” and “was *buildin’*” instead of simply “*building*” and “*buildin’*”), thereby forcing a progressive interpretation. This experiment aimed to determine whether resolving part-of-speech ambiguity by forcing a progressive interpretation would improve the correlation between elicited and corpus word biases.

#### Participants

A total of 30 participants were recruited from Prolific to participate in this follow-up rating task, ensuring a sample size of 30 ratings per word. Participants followed the same general procedure outlined in Section “General methodology”. However, unlike Experiments 1 and 2, which used 318 words divided into lists, this experiment used only the 68 PNC words presented in a single list to all participants. It is true that Experiment 3 relies on a smaller sample of items. This move was made so that we could focus exclusively on the items for which we had reliable corpus data. In this task, participants rated items such as “was *building*” and “was *buildin’*” instead of simply “*building*” and “*buildin’*”. As in the previous experiments, three catch trials were included to ensure attention. These were adapted to the context of the study (e.g., “was *bringing*” vs. “was *bringin’*”).

**Fig. 10:**
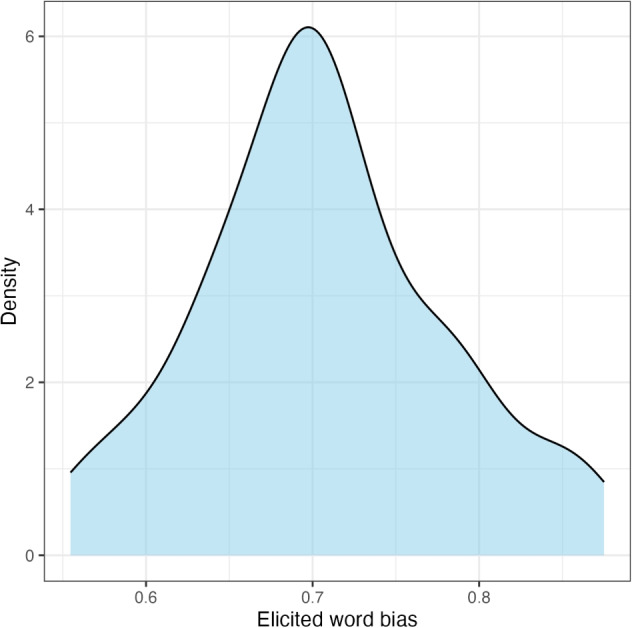
Experiment 3: Distribution of elicited word biases

**Fig. 11:**
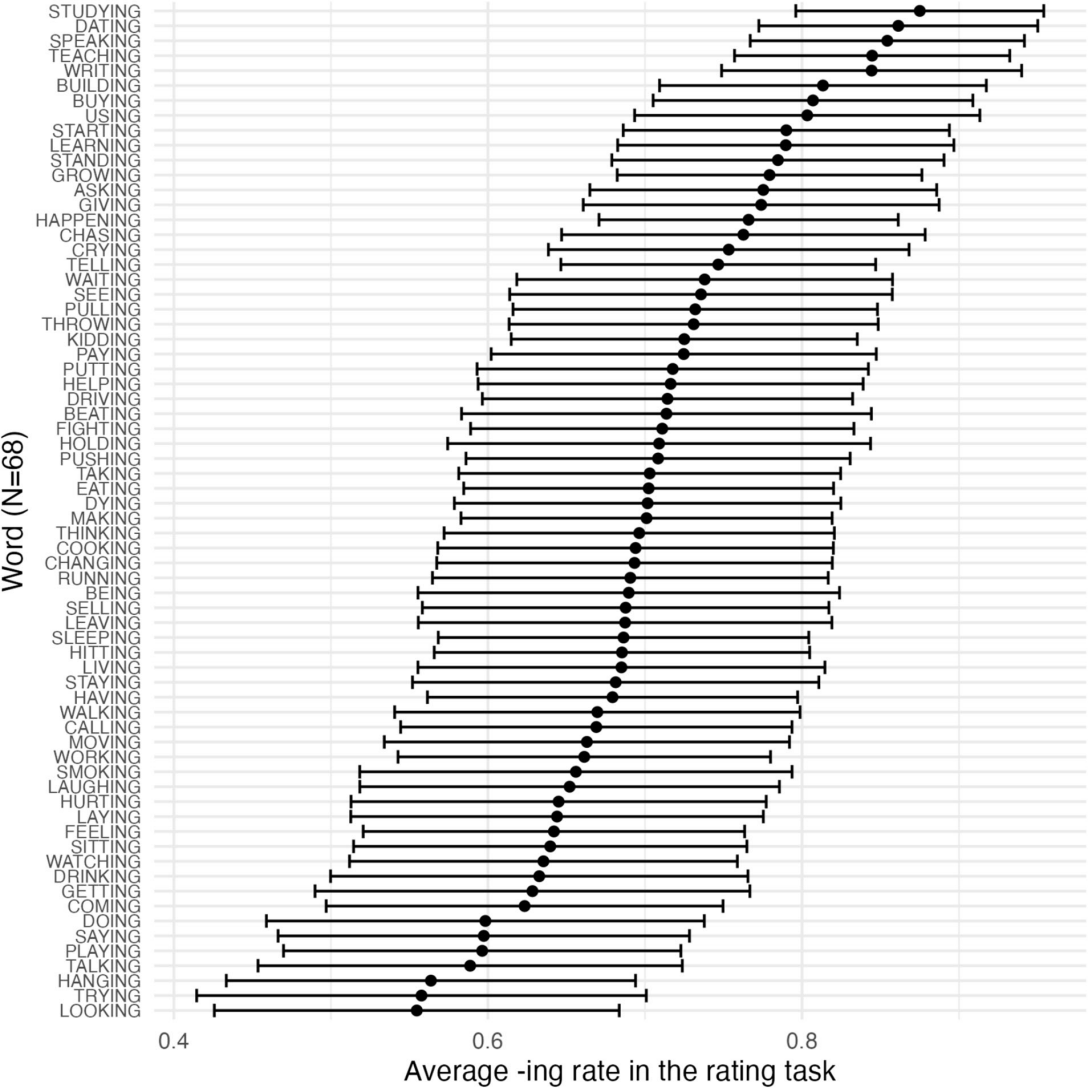
Experiment 3: Word-specific confidence intervals for the 68 ING words taken from PNC

While presenting an “auxiliary + V-ING" fragment on its own is admittedly artificial, this approach is not obviously less natural than rating words in isolation. Crucially, it avoids the numerous confounds that would arise from using full sentences, such as potential associations between subject gender and the two variants, stylistic covariation induced by copula contraction or lack thereof, and many more. Furthermore, if the objective is to rate verbs as they appear in the progressive construction, then the collocational relationship between the auxiliary and the verb is a deliberate and central feature of the design. Figure 10 shows the raw distribution of elicited word biases for the 68 ING words in Experiment 3. The mean rating and 95% confidence interval for each of the 68 words used in Experiment 3 are plotted below in Fig. 11.

#### Analysis and results

Participants who failed the catch trials were excluded from analysis (*N* = 0), resulting in 0 exclusions.

**Fig. 12:**
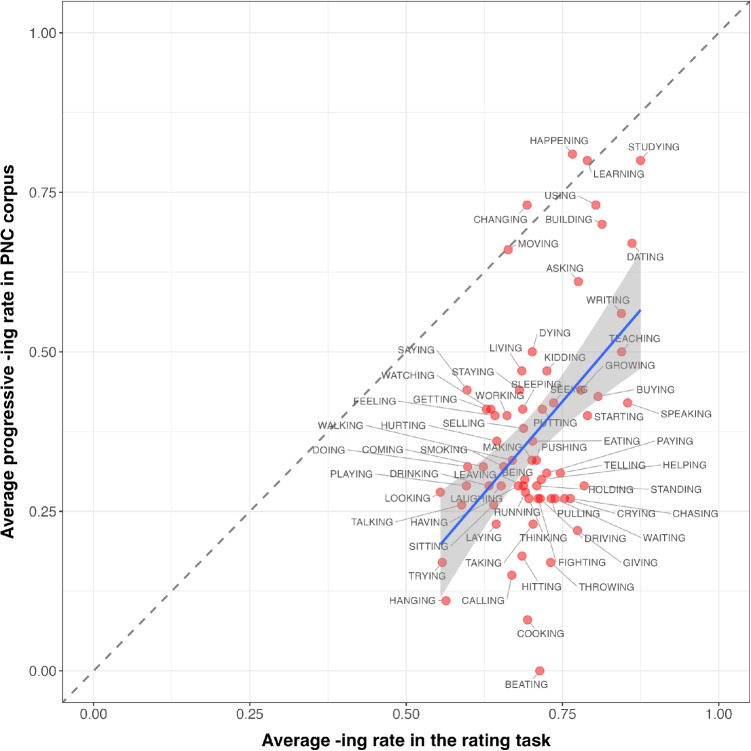
Correlations between corpus word bias and elicited word bias for words in progressive contexts

Pearson correlation yielded a stronger correlation coefficient (Pearson’s R = 0.49, $p<$ 0.001), as can be seen in Fig. 12. This further indicates that part-of-speech ambiguity is a significant factor that should be taken into consideration for future research on this topic, perhaps inviting refinement of the rating task.

## General discussion

This study empirically validates whether self-reported estimates of sociolinguistic variant use reliably reflect real-world patterns by analyzing correlations between elicited measures and conversational speech data for the English phonological variable ING. Our results reveal a statistically significant, yet modest, positive correlation, as well as some asymmetries in the relationship between corpus and elicited word biases. This suggests that while speakers can report broad tendencies in word bias distributions, these preferences do not consistently mirror precise usage patterns, and may also be shaped by other kinds of biases.

Therefore, rather than validating rating tasks as a simple proxy for corpus patterns, our findings highlight the complex nature of such tasks. Rating tasks are not merely reflecting actual production, but appear to tap into a different level of linguistic knowledge, raising critical questions about how speakers evaluate single-word prompts in the absence of context. This discrepancy underscores that elicited judgments and corpus data should not be seen as interchangeable. This does not invalidate the use of rating tasks; rather, it calls for a nuanced understanding of their limitations. Such tasks provide valuable proxies for certain linguistic behaviors, but it is important to bear in mind that their outputs are susceptible to significant noise. We also acknowledge that corpus data does not serve as a perfect “ground truth”, since naturalistic data is also subject to its own sources of noise and bias. Neither method, therefore, offers a flawless answer to what is being examined here. Researchers who use rating tasks should be aware of the specific constructs being measured, rather than treating their results as a direct correspondence to corpus data. Our work here showcases a preliminary step in generating more robust word-bias norms, and we advocate for continued research to refine these methodologies.

This being said, the positive correlations between elicited word biases and corpus word biases further suggest that speakers do track and possess some degree of implicit knowledge of word biases of variable ING, which they can report in experimental contexts. These findings further highlight the value of incorporating word bias into studies of sociolinguistic variation. The present analysis examined 318 ING words and elicited the word biases for all of these words. While these data, provided in the Appendix A, may be of interest to researchers studying how sociolinguistic variation is perceived and produced, they should also be treated with due awareness of the specific methods used to collect them.

Regarding this aspect, our results point to a crucial methodological takeaway: elicited word biases generated from words in isolation are less reliable proxies for real-world production patterns compared to elicited biases of words presented with even minimal syntactic context (i.e., the presence of an auxiliary verb). While it is tempting to extend this observation to conclude that future work should *maximize* contextual information, such as by presenting target words in full sentences or even short dialogues, that approach would introduce a significant trade-off. As soon as a target word is embedded in a fuller context, properties of that specific context—such as the semantics of the sentences or the pragmatics of the dialogue—have the potential to influence judgments in ways that are difficult to disentangle from the properties of the target word itself. Our use of a minimal syntactic context was therefore a deliberate methodological choice, intended to strike a balance: providing enough syntactic frame to force a part-of-speech interpretation and thus improve on de-contextualized ratings, while minimizing the introduction of additional confounding factors. Future work can build on this finding by systematically exploring this trade-off, for instance, by comparing minimal contexts to richer carrier sentences to better understand which contextual factors people are most sensitive to in these rating tasks. We suggest that such next steps are most likely to be fruitful if they continue to ask how rating differences compare to corpus patterns of word-specificity.

Our findings also further add to the line of work on subjective frequency measures in psycholinguistic research. When estimating the frequency of monosyllabic words, subjective frequency ratings—where individuals estimate how often they have encountered a given word—have been found to correlate well with objective log frequency estimates—for both spoken and written words, as well as for spoken and sign languages (Balota et al., 2001; Ferrand et al., 2008; Mayberry et al., 2014; Thompson & Desrochers, 2009). Different from familiarity ratings, which are contingent on how well individuals know a given word, subjective frequency measures are also better predictors for lexical processing (Balota et al., 2001). In cases where the ratings involve syntactic choices, speakers demonstrate implicit knowledge of their quantitative usage patterns, and their subjective naturalness ratings align with corpus model predictions (Bresnan et al., 2007). We further show that speakers can report how often a pronunciation variant tends to co-occur with a particular word, and the subjective frequency of this kind also correlates relatively well with conversational corpus data. However, it still remains unclear what the true nature is for these word biases. Even though we are not in a position to fully answer this question, our results can shed light on how variable pronunciations are represented in the mental lexicon.

In our current analysis comparing the two prompts, we found that the one emphasizing individual-level patterns did not produce estimates significantly different from those elicited by the prompt focusing on community-level expectations. It is possible that the conversational context is more important for eliciting these kinds of word biases. Since the context has been fixed to be “conversations with friends”, participants may mainly rely on the context rather than the question specifics to retrieve their estimates. Moreover, previous work on how ING is conditioned primarily analyzed patterns aggregated over conversational speech data within speech communities. Despite the fact that there exists individual-level variability, uniformity may exist across individual speakers. Therefore, the different prompts might ultimately get at the same estimates. However, it should not be assumed that individual production and community-level expectations always match (Forrest, 2015).

It is also important to note that our corpus data represented a regional variety of American English, and participants were recruited via crowd-sourcing without strict control over their dialectal backgrounds. This has two key methodological implications, given that the variable ING is sensitive to dialectal differences. First, speakers’ exposure to the two variants—*-in’* and*-ing*—may vary across dialects, meaning that their estimated word biases could reflect differing linguistic experiences. Second, corpus word biases themselves may also differ if another regional variety were analyzed. Consequently, the strength of correlations between elicited and corpus word biases could vary across dialects. Future research should examine the extent of dialectal influence on these correlations. Finally, while our validation is based on a single case study of variable ING, future research is needed to determine whether these correlations extend to other cases of pronunciation variability. The ratings provided here (see Appendix A) for a total of 318 ING-containing words can thus serve as a reference dataset for future work on pronunciation variation, either for this specific phenomenon or others.

Finally, our validation of experimentally elicited word bias measures opens up several promising avenues for future research on language variation. First, word biases, or, more broadly, variant relative frequencies, could offer valuable insights to studies on sociolinguistic perception and evaluation. For instance, while it is well established that linguistic variants carry distinct social meanings (Campbell-Kibler, 2011), it remains unclear whether this evaluation is conditioned by the specific word in which a variant appears, given that not all tokens likely carry the same social weight. It is possible that a speaker who produces a non-standard variant in a word that strongly favors the standard form (e.g., saying *studyin’* instead of *studying*) would be perceived differently than a speaker who uses the same variant in a word where it is more common (e.g., *kickin’*). The violation of the expected word-specific norm may trigger stronger or different social evaluations, such as being perceived as more informal, more masculine, or more authentic. To empirically test this idea, one could make use of words with different biases toward a particular variant to see how word-specific expectations modulate the social meaning attributed to speakers. Such an approach could be of value in understanding how listeners’ evaluations arise from the complex interplay of social and cognitive payoffs and costs in real-time interaction (Sharma, 2025). Beyond the phenomenon of sociolinguistic variation, these word bias estimates can also be incorporated more broadly to understand word-level variability involved in language processing in general.

Although this study demonstrates the methodology using ING—a stable sociolinguistic variable with a well-established orthographic representation—this approach holds promise for a wider range of variable phenomena. Specifically, we believe this method could successfully be extended to any variable whose variants can be represented orthographically, such as word-final t/d deletion and third-person singular “-s” absence. However, it is also worth acknowledging that the claims we are making here in this paper may not be applicable to all variables. While this text-based format seems less suitable for continuous phonetic variation (e.g., vowel quality), the rating paradigm could potentially be adapted for use with auditory stimuli. Therefore, a potential next step would be to assess whether a similar approach can be effective with auditory presentation.

By improving our understanding of methods for quantifying word biases, we hope to enable psycholinguists and sociolinguists to ask new questions about the linguistic and social forces that shape language variation and change. This approach moves the field beyond treating all tokens of a variable as socially or structurally equivalent and toward a more integrated model of language use and social perception.

## Data Availability

Data and scripts relevant to this study are available at https://osf.io/m8bqh.

## Appendix A

Elicited word bias (under different prompts and contexts) and corpus word bias of 318 ING-suffixed words. Bias scores for each word were calculated using ratings from approximately 30 participants.

**Table:**

Word	Elicited word bias	Elicited word bias	Elicited word bias	Word bias
	(from production)	(from expectations)	(with context)	(corpus)
ASKING	0.53	0.65	0.76	0.61
BAKING	0.8	0.69	NA	NA
BASKING	0.75	0.81	NA	NA
BASTING	0.76	0.87	NA	NA
BEATING	0.75	0.58	0.71	0
BEEPING	0.74	0.65	NA	NA
BEGGING	0.66	0.62	NA	NA
BEING	0.75	0.72	0.69	0.3
BELCHING	0.81	0.63	NA	NA
BENDING	0.78	0.68	NA	NA
BIKING	0.79	0.79	NA	NA
BLAMING	0.8	0.63	NA	NA
BLEETING	0.63	0.64	NA	NA
BLINKING	0.81	0.62	NA	NA
BLOCKING	0.7	0.68	NA	NA
BLOGGING	0.79	0.81	NA	NA
BLOOMING	0.77	0.60	NA	NA
BLUFFING	0.83	0.57	NA	NA
BLUSHING	0.8	0.71	NA	NA
BOASTING	0.78	0.74	NA	NA
BOUNCING	0.59	0.61	NA	NA
BRAGGING	0.68	0.54	NA	NA
BRAWLING	0.7	0.59	NA	NA
BREACHING	0.86	0.82	NA	NA
BREAKING	0.7	0.65	NA	NA
BREATHING	0.73	0.73	NA	NA
BREWING	0.81	0.54	NA	NA
BRIBING	0.89	0.81	NA	NA
BRUSHING	0.71	0.54	NA	NA
BUDGING	0.75	0.76	NA	NA
BUILDING	0.77	0.57	0.81	0.7
BUMPING	0.67	0.56	NA	NA
BURNING	0.75	0.65	NA	NA
BURPING	0.83	0.73	NA	NA
BURSTING	0.75	0.63	NA	NA
BUYING	0.59	0.66	0.81	0.43
CALLING	0.67	0.62	0.67	0.15
CARVING	0.76	0.80	NA	NA
CHANGING	0.84	0.69	0.69	0.73
CHARRING	0.82	0.86	NA	NA
CHASING	0.73	0.70	0.76	0.27
CHATTING	0.72	0.53	NA	NA
CHEERING	0.73	0.74	NA	NA
CHEWING	0.67	0.50	NA	NA
CLANGING	0.64	0.86	NA	NA
CLAPPING	0.73	0.70	NA	NA
CLASHING	0.94	0.74	NA	NA
CLAWING	0.76	0.75	NA	NA
CLEANING	0.64	0.64	NA	NA
CLICKING	0.67	0.75	NA	NA
CLIMBING	0.69	0.77	NA	NA
CLIPPING	0.82	0.65	NA	NA
CLOAKING	0.89	0.85	NA	NA
CLOGGING	0.72	0.68	NA	NA
CLOSING	0.66	0.78	NA	NA
CLUCKING	0.64	0.73	NA	NA
CLUTCHING	0.87	0.67	NA	NA
COMBING	0.85	0.83	NA	NA
COMING	0.56	0.35	0.62	0.32
COOKING	0.58	0.58	0.69	0.08
COPING	0.88	0.84	NA	NA
COUGHING	0.57	0.70	NA	NA
COUNTING	0.77	0.62	NA	NA
CRAFTING	0.94	0.77	NA	NA
CRAMMING	0.7	0.75	NA	NA
CRAWLING	0.72	0.48	NA	NA
CREEPING	0.46	0.60	NA	NA
CROAKING	0.71	0.81	NA	NA
CROONING	0.9	0.78	NA	NA
CRYING	0.6	0.58	0.75	0.27
DATING	0.95	0.85	0.86	0.67
DELVING	0.82	0.85	NA	NA
DINING	0.81	0.78	NA	NA
DOCKING	0.74	0.80	NA	NA
DOING	0.52	0.41	0.6	0.32
DOTTING	0.84	0.71	NA	NA
DOZING	0.74	0.57	NA	NA
DRAINING	0.76	0.86	NA	NA
DREAMING	0.72	0.60	NA	NA
DRINKING	0.63	0.44	0.63	0.29
DRIPPING	0.56	0.68	NA	NA
DRIVING	0.58	0.73	0.71	0.27
DROOLING	0.68	0.72	NA	NA
DROOPING	0.82	0.61	NA	NA
DROPPING	0.68	0.61	NA	NA
DROWNING	0.77	0.55	NA	NA
DRUMMING	0.61	0.80	NA	NA
DYING	0.53	0.59	0.7	0.5
EATING	0.81	0.52	0.7	0.36
FAKING	0.65	0.65	NA	NA
FARMING	0.89	0.70	NA	NA
FEELING	0.6	0.54	0.64	0.4
FENDING	0.89	0.85	NA	NA
FIGHTING	0.64	0.54	0.71	0.27
FILMING	0.8	0.67	NA	NA
FINDING	0.7	0.76	NA	NA
FIXING	0.56	0.60	NA	NA
FLAMING	0.61	0.52	NA	NA
FLAPPING	0.65	0.60	NA	NA
FLARING	0.82	0.65	NA	NA
FLICKING	0.75	0.75	NA	NA
FLOCKING	0.81	0.79	NA	NA
FLOPPING	0.7	0.45	NA	NA
FLOWING	0.75	0.74	NA	NA
FOAMING	0.82	0.64	NA	NA
FOLDING	0.68	0.73	NA	NA
FORGING	0.86	0.81	NA	NA
FREEZING	0.75	0.49	NA	NA
FROWNING	0.83	0.77	NA	NA
FUSSING	0.62	0.39	NA	NA
GAINING	0.73	0.70	NA	NA
GASPING	0.75	0.78	NA	NA
GETTING	0.46	0.53	0.63	0.41
GIVING	0.76	0.62	0.77	0.22
GLARING	0.87	0.82	NA	NA
GLEAMING	0.83	0.75	NA	NA
GLIDING	0.85	0.64	NA	NA
GLOWING	0.74	0.66	NA	NA
GNAWING	0.63	0.74	NA	NA
GRABBING	0.68	0.54	NA	NA
GRINNING	0.7	0.69	NA	NA
GROWING	0.78	0.56	0.78	0.44
GUESSING	0.81	0.51	NA	NA
GULPING	0.78	0.76	NA	NA
HANGING	0.59	0.43	0.56	0.11
HAPPENING	0.68	0.44	0.77	0.81
HAVING	0.56	0.67	0.68	0.29
HELPING	0.69	0.69	0.72	0.3
HIKING	0.8	0.70	NA	NA
HITTING	0.7	0.52	0.69	0.18
HOARDING	0.89	0.76	NA	NA
HOISTING	0.83	0.85	NA	NA
HOLDING	0.57	0.65	0.71	0.29
HOPING	0.72	0.46	NA	NA
HOSTING	0.77	0.88	NA	NA
HUGGING	0.62	0.66	NA	NA
HURTING	0.6	0.51	0.65	0.36
ITCHING	0.64	0.52	NA	NA
JABBING	0.75	0.67	NA	NA
JIVING	0.62	0.40	NA	NA
JOGGING	0.64	0.67	NA	NA
JOINING	0.75	0.73	NA	NA
JOKING	0.66	0.47	NA	NA
JOUSTING	0.88	0.82	NA	NA
JUDGING	0.77	0.69	NA	NA
JUMPING	0.5	0.71	NA	NA
KIDDING	0.66	0.65	0.73	0.47
KNOCKING	0.57	0.65	NA	NA
LACKING	0.74	0.75	NA	NA
LAGGING	0.84	0.72	NA	NA
LAUGHING	0.56	0.61	0.65	0.29
LAUNCHING	0.66	0.68	NA	NA
LAYING	0.62	0.44	0.64	0.23
LEAPING	0.71	0.61	NA	NA
LEARNING	0.77	0.73	0.79	0.8
LEAVING	0.59	0.44	0.69	0.29
LENDING	0.79	0.80	NA	NA
LIMPING	0.77	0.74	NA	NA
LINKING	0.85	0.69	NA	NA
LIVING	0.53	0.53	0.69	0.47
LOOKING	0.43	0.56	0.55	0.28
LOSING	0.73	0.66	NA	NA
LOVING	0.68	0.46	NA	NA
LUGGING	0.73	0.72	NA	NA
LURCHING	0.71	0.82	NA	NA
MAKING	0.73	0.44	0.7	0.33
MENDING	0.94	0.71	NA	NA
MERGING	0.89	0.77	NA	NA
MESSING	0.56	0.39	NA	NA
MIXING	0.61	0.74	NA	NA
MOPPING	0.61	0.64	NA	NA
MOVING	0.67	0.63	0.66	0.66
MOWING	0.66	0.67	NA	NA
MUNCHING	0.56	0.48	NA	NA
NAPPING	0.77	0.61	NA	NA
NUDGING	0.73	0.84	NA	NA
PASTING	0.85	0.87	NA	NA
PATCHING	0.83	0.66	NA	NA
PAYING	0.69	0.58	0.72	0.31
PECKING	0.75	0.67	NA	NA
PEEPING	0.73	0.51	NA	NA
PICKING	0.58	0.68	NA	NA
PINCHING	0.77	0.78	NA	NA
PLAYING	0.47	0.52	0.6	0.29
PLEADING	0.89	0.73	NA	NA
PLOWING	0.72	0.75	NA	NA
POUNCING	0.82	0.65	NA	NA
PREACHING	0.59	0.62	NA	NA
PROBING	0.86	0.82	NA	NA
PRODDING	0.89	0.62	NA	NA
PROWLING	0.74	0.56	NA	NA
PRYING	0.81	0.63	NA	NA
PULLING	0.68	0.56	0.73	0.27
PUMPING	0.61	0.60	NA	NA
PUSHING	0.65	0.53	0.71	0.33
PUTTING	0.64	0.66	0.72	0.41
QUAKING	0.75	0.81	NA	NA
RAKING	0.75	0.76	NA	NA
REACHING	0.69	0.64	NA	NA
REEKING	0.82	0.67	NA	NA
REVVING	0.5	0.79	NA	NA
RHYMING	0.65	0.69	NA	NA
RISING	0.91	0.76	NA	NA
RISKING	0.86	0.70	NA	NA
ROARING	0.72	0.56	NA	NA
RUNNING	0.68	0.87	0.69	0.28
RUINING	0.68	0.44	NA	NA
SAGGING	0.7	0.65	NA	NA
SAVING	0.87	0.68	NA	NA
SAYING	0.44	0.52	0.6	0.44
SCANNING	0.81	0.75	NA	NA
SCARING	0.72	0.67	NA	NA
SCHMOOZING	0.59	0.71	NA	NA
SCORING	0.74	0.75	NA	NA
SCOWLING	0.84	0.66	NA	NA
SEALING	0.84	0.85	NA	NA
SEARCHING	0.73	0.61	NA	NA
SEEING	0.59	0.66	0.74	0.42
SEEPING	0.71	0.67	NA	NA
SELLING	0.79	0.62	0.69	0.38
SENDING	0.69	0.73	NA	NA
SENSING	0.95	0.75	NA	NA
SHINING	0.82	0.74	NA	NA
SHIPPING	0.78	0.77	NA	NA
SHOPPING	0.56	0.62	NA	NA
SHOUTING	0.7	0.80	NA	NA
SHOVING	0.75	0.53	NA	NA
SHRUGGING	0.74	0.71	NA	NA
SHUNNING	0.8	0.80	NA	NA
SIGHING	0.83	0.85	NA	NA
SINGING	0.52	0.69	NA	NA
SIPPING	0.65	0.44	NA	NA
SITTING	0.68	0.59	0.64	0.26
SKIDDING	0.74	0.64	NA	NA
SKIMMING	0.77	0.62	NA	NA
SKIPPING	0.6	0.65	NA	NA
SLAMMIG	0.55	0.54	NA	NA
SLEEPING	0.66	0.48	0.69	0.41
SLIPPING	0.68	0.55	NA	NA
SMEARING	0.73	0.80	NA	NA
SMILING	0.69	0.56	NA	NA
SMIRKING	0.69	0.77	NA	NA
SMOKING	0.49	0.56	0.66	0.32
SNACKING	0.57	0.57	NA	NA
SPEAKING	0.67	0.50	0.85	0.42
SNEERING	0.86	0.80	NA	NA
SNEEZING	0.84	0.64	NA	NA
SNOOPING	0.51	0.70	NA	NA
SNOOZING	0.54	0.58	NA	NA
SNOWING	0.68	0.56	NA	NA
SOAKING	0.77	0.71	NA	NA
SOBBING	0.82	0.74	NA	NA
SPAWNING	0.83	0.76	NA	NA
SPEAKING	0.66	0.81	NA	NA
SPEEDING	0.64	0.77	NA	NA
SPINNING	0.74	0.53	NA	NA
SPRAYING	0.8	0.69	NA	NA
STACKING	0.72	0.52	NA	NA
STANDING	0.73	0.65	0.78	0.29
STARTING	0.74	0.61	0.79	0.4
STAYING	0.65	0.32	0.68	0.44
STEALING	0.6	0.73	NA	NA
STEAMING	0.66	0.70	NA	NA
STEERING	0.83	0.78	NA	NA
STEPPING	0.64	0.61	NA	NA
STICKING	0.72	0.41	NA	NA
STITCHING	0.74	0.69	NA	NA
STOPPING	0.59	0.61	NA	NA
STRAYING	0.77	0.54	NA	NA
STUDYING	0.75	0.75	0.88	0.8
SURGING	0.85	0.88	NA	NA
SWAPPING	0.76	0.65	NA	NA
SWEEPING	0.6	0.65	NA	NA
SWIMMING	0.6	0.74	NA	NA
SWIRLING	0.82	0.57	NA	NA
SWITCHING	0.7	0.64	NA	NA
SWOONING	0.86	0.65	NA	NA
TAKING	0.59	0.67	0.7	0.23
TALKING	0.63	0.48	0.59	0.26
TAMING	0.9	0.84	NA	NA
TANNING	0.81	0.87	NA	NA
TEACHING	0.76	0.76	0.84	0.5
TELLING	0.66	0.42	0.75	0.31
THIEVING	0.74	0.60	NA	NA
THINKING	0.56	0.63	0.7	0.27
THRIVING	0.82	0.85	NA	NA
THROWING	0.66	0.69	0.73	0.17
THUMPING	0.75	0.53	NA	NA
TIDING	0.89	0.89	NA	NA
TRASHING	0.7	0.71	NA	NA
TRUCKING	0.67	0.37	NA	NA
TRYING	0.66	0.42	0.56	0.17
TWEAKING	0.74	0.46	NA	NA
TWIRLING	0.78	0.55	NA	NA
TWITCHING	0.68	0.61	NA	NA
USING	0.74	0.65	0.8	0.73
VOUCHING	0.82	0.88	NA	NA
WAITING	0.69	0.45	0.74	0.27
WALKING	0.46	0.58	0.67	0.33
WALTZING	0.87	0.80	NA	NA
WASHING	0.74	0.74	NA	NA
WASTING	0.77	0.69	NA	NA
WATCHING	0.55	0.56	0.64	0.41
WAXING	0.81	0.84	NA	NA
WEEPING	0.75	0.87	NA	NA
WHACKING	0.74	0.44	NA	NA
WHIRLING	0.75	0.67	NA	NA
WHOOPING	0.65	0.37	NA	NA
WIELDING	0.78	0.84	NA	NA
WINKING	0.78	0.78	NA	NA
WINNING	0.9	0.59	NA	NA
WISHING	0.68	0.61	NA	NA
WORKING	0.63	0.49	0.66	0.4
WRITING	0.82	0.83	0.84	0.56
YEARNING	0.86	0.68	NA	NA
YELLING	0.56	0.65	NA	NA
YIELDING	0.84	0.87	NA	NA
ZAPPING	0.77	0.49	NA	NA

## Footnotes

1 We thank our reviewers for pointing this out.

## Code availability

Analysis code for this study is available at https://osf.io/m8bqh.
